# Supplementary material for: A deep population reference panel of tandem repeat variation
Source: Nat Commun. 2023 Oct 23;14:6711. doi: 10.1038/s41467-023-42278-3 (PMC10593948; doi:10.1038/s41467-023-42278-3)
Supplement: Supplementary file 3 — Description of Additional Supplementary Files [file 41467_2023_42278_MOESM3_ESM.pdf]

Description of Additional Supplementary Files

File Name: Supplementary Data 1
Description: Impact of TR composition on Mendelian Inheritance

File Name: Supplementary Data 2
Description: Evaluating MI at TRs called by multiple methods

File Name: Supplementary Data 3
Description: Primers used for PCR validation of individual TR loci

File Name: Supplementary Data 4
Description: Product sizes (in bp) from PCR validation of TR genotypes

File Name: Supplementary Data 5
Description: Genotypes obtained from Asuragen for C9orf72, HTT, and FMR1

File Name: Supplementary Data 6
Description: Comparison of WGS based genotypes to those obtained from capillary electrophoresis

File Name: Supplementary Data 7
Description: Binsets used to convert product sizes to genotypes comparable to WGS-based calls

File Name: Supplementary Data 8
Description: Summary of TR variation in each sample

File Name: Supplementary Data 9
Description: Summary of variation at protein-coding TRs

File Name: Supplementary Data 10
Description: TRs for which most samples do not match hg38

File Name: Supplementary Data 11
Description: Candidate population-specific TR expansions

File Name: Supplementary Data 12
Description: Genotypes at candidate TR expansions in CA10 and near NEXN based on manual inspection of Pacbio Hifi reads.

File Name: Supplementary Data 13
Description: Spearman correlation of sequence features with TR heterozygosity

File Name: Supplementary Data 14
Description: Significant eTRs in EUR

File Name: Supplementary Data 15
Description: Significant eTRs in AFR

File Name: Supplementary Data 16
Description: Pearson correlation (r) between eTR effect sizes from Geuvadis samples and effect sizes computed in GTEx in Fotsing et al.

File Name: Supplementary Data 17
Description: Imputation concordance of trait-associated loci.

File Name: Supplementary Data 18
Description: Best tag SNP for each TR per population
