# Supplementary material for: A deep population reference panel of tandem repeat variation
Source: Nat Commun. 2023 Oct 23;14:6711. doi: 10.1038/s41467-023-42278-3 (PMC10593948; doi:10.1038/s41467-023-42278-3)
Supplement: Supplementary file 6 — Reporting Summary [file 41467_2023_42278_MOESM6_ESM.pdf]

nature portfolio

Corresponding author(s): Melissa Gymrek

Last updated by author(s): Sep 22, 2023

Reporting Summary

Nature Portfolio wishes to improve the reproducibility of the work that we publish. This form provides structure for consistency and transparency in reporting. For further information on Nature Portfolio policies, see our Editorial Policies and the Editorial Policy Checklist.

Statistics

For all statistical analyses, confirm that the following items are present in the figure legend, table legend, main text, or Methods section.

n/a	Confirmed	
☐	☒	The exact sample size (*n*) for each experimental group/condition, given as a discrete number and unit of measurement
☐	☒	A statement on whether measurements were taken from distinct samples or whether the same sample was measured repeatedly
☒	☐	The statistical test(s) used AND whether they are one- or two-sided<br>*Only common tests should be described solely by name; describe more complex techniques in the Methods section.*
☐	☒	A description of all covariates tested
☐	☒	A description of any assumptions or corrections, such as tests of normality and adjustment for multiple comparisons
☐	☒	A full description of the statistical parameters including central tendency (e.g. means) or other basic estimates (e.g. regression coefficient) AND variation (e.g. standard deviation) or associated estimates of uncertainty (e.g. confidence intervals)
☐	☒	For null hypothesis testing, the test statistic (e.g. *F*, *t*, *r*) with confidence intervals, effect sizes, degrees of freedom and *P* value noted<br>*Give P values as exact values whenever suitable.*
☒	☐	For Bayesian analysis, information on the choice of priors and Markov chain Monte Carlo settings
☒	☐	For hierarchical and complex designs, identification of the appropriate level for tests and full reporting of outcomes
☐	☒	Estimates of effect sizes (e.g. Cohen's *d*, Pearson's *r*), indicating how they were calculated

Our web collection on statistics for biologists contains articles on many of the points above.

Software and code

Policy information about availability of computer code

Data collection	No software was used for data collection.
Data analysis	HipSTR v0.6.2<br>adVNTR v1.4.0<br>ExpansionHunter v5.0.0<br>GangSTR v2.4.5<br>TRTools v4.0.1<br>samtools v1.5<br>BLASTN v2.13.0<br>python scikit-learn library v1.0.2<br>STRetch v.0.4.0<br>ExpansionHunter Denovo v.0.9.0<br>HOMER v4.11.1<br>smartpca v.13050<br>ANNOVAR<br>plink v.1.90b3.44<br>Beagle v5.4 and v4.0<br>OrganismDbi R package v1.40.0<br>TRviz v1.0.1<br>python scipy library v1.5.2

PEER v1.0
EnsembleTR v1.0.0 (developed here): https://github.com/gymrek-lab/EnsembleTR

For manuscripts utilizing custom algorithms or software that are central to the research but not yet described in published literature, software must be made available to editors and reviewers. We strongly encourage code deposition in a community repository (e.g. GitHub). See the Nature Portfolio guidelines for submitting code & software for further information.

Data

Policy information about availability of data

All manuscripts must include a data availability statement. This statement should provide the following information, where applicable:

- Accession codes, unique identifiers, or web links for publicly available datasets
- A description of any restrictions on data availability
- For clinical datasets or third party data, please ensure that the statement adheres to our policy

TR genotypes, the phased TR-SNP reference panel, and population-specific summary statistics generated in this study are available from the EnsembleTR Github webpage [https://github.com/gymrek-lab/EnsembleTR]. Summary statistics are also made available in browsable format at WebSTR [webstr.ucsd.edu]. Summary statistics of all tested TR-gene pairs in African and European samples are deposited in the Figshare database [https://figshare.com/articles/dataset/1000GenomesH3Africa_SuppData16_zip/24164367].

The WGS datasets for the 1000GP samples used in this study are available from the European Nucleotide Archive under accessions PRJEB31736 [https://www.ebi.ac.uk/ena/browser/view/PRJEB31736] (unrelated samples) and PRJEB36890 [https://www.ebi.ac.uk/ena/browser/view/PRJEB36890] (related samples). The H3Africa WGS datasets used in this study are available in the European Genome-Phenome Archive under accession EGAS00001002976 [https://ega-archive.org/studies/EGAS00001002976/]. Geuvadis datasets used in this study are available from the 1000GP website [https://www.internationalgenome.org/data-portal/data-collection/geuvadis]. Analyses are based on the GRCh38 reference genome [https://storage.googleapis.com/genomics-public-data/resources/broad/hg38/v0/Homo_sapiens_assembly38.fasta].

Research involving human participants, their data, or biological material

Policy information about studies with human participants or human data. See also policy information about sex, gender (identity/presentation), and sexual orientation and race, ethnicity and racism.

Reporting on sex and gender	N/A
Reporting on race, ethnicity, or other socially relevant groupings	N/A
Population characteristics	N/A
Recruitment	N/A
Ethics oversight	N/A

Note that full information on the approval of the study protocol must also be provided in the manuscript.

Field-specific reporting

Please select the one below that is the best fit for your research. If you are not sure, read the appropriate sections before making your selection.

☒ Life sciences ☐ Behavioural & social sciences ☐ Ecological, evolutionary & environmental sciences

For a reference copy of the document with all sections, see nature.com/documents/nr-reporting-summary-flat.pdf

Life sciences study design

All studies must disclose on these points even when the disclosure is negative.

Sample size	No sample size calculation was performed. We used the sample size available for the 1000 Genomes Project (3203 samples) and H3Africa (348 samples). In total we had 3550 samples in our analysis. This sample size is sufficient to characterize the majority of common TR alleles in the populations analyzed.
Data exclusions	We did not exclude any sample.
Replication	We compared population structure and population specific allele frequencies to previously published results. We validated a subset of calls by comparing them to results from fragment analysis via capillary electrophoresis on the same samples. Only one replicate of capillary electrophoresis was performed
Randomization	Randomization is not relevant to this study. Our study does not use any case/control labels. Analyses where replication would typically be

Randomization	used (e.g. trials) were not performed here
Blinding	Blinding is not relevant to this study. Our study does not use any case/control labels. Analyses where blinding would typically be used (e.g. trials) were not performed here.

Reporting for specific materials, systems and methods

We require information from authors about some types of materials, experimental systems and methods used in many studies. Here, indicate whether each material, system or method listed is relevant to your study. If you are not sure if a list item applies to your research, read the appropriate section before selecting a response.

Materials & experimental systems

n/a	Involved in the study
☒	☐ Antibodies
☒	☐ Eukaryotic cell lines
☒	☐ Palaeontology and archaeology
☒	☐ Animals and other organisms
☒	☐ Clinical data
☒	☐ Dual use research of concern
☒	☐ Plants

Methods

n/a	Involved in the study
☒	☐ ChIP-seq
☒	☐ Flow cytometry
☒	☐ MRI-based neuroimaging
